# SH2 domain‐containing protein tyrosine phosphatase‐2 is enriched in eyelid specimens of rosacea

**DOI:** 10.1002/ski2.190

**Published:** 2022-11-17

**Authors:** Apoorv Chebolu, Ramon Bossardi Ramos, Thilaka Arunachalam, Alejandro Pablo Adam, Edward J. Wladis

**Affiliations:** ^1^ Department of Ophthalmology Lions Eye Institute Albany New York USA; ^2^ Department of Molecular and Cellular Physiology Albany Medical Center Albany New York USA

## Abstract

**Background:**

Rosacea is a cutaneous disease that may secondarily affect the ocular surface. Due to the vision threatening, cosmetic, psychological, and work productivity impact, the identification of cellular targets that govern rosacea would enhance our understanding of the biology of the disease and delineate targets for therapeutic manipulation.

**Objective:**

To characterize the involvement of SH2 domain‐containing protein tyrosine phosphatase‐2 (SHP2) in the pathogenesis of rosacea.

**Methods:**

Specimens from elective ectropion surgery (*n* = 20) were processed from patients with rosacea (*n* = 10) and control patients (*n* = 10). Immunohistochemistry (IHC) and quantitative western blotting (WB) were performed to identify and quantify the presence of SHP2 and 4G10 (a phosphotyrosine antibody) in rosacea compared to normal tissue. IHC samples were graded according to an intensity scale (0–4). Mann‐Whitney statistical analyses were performed via a dedicated computerized software package.

**Results:**

On WB, SHP2 was expressed in higher concentrations in rosacea specimens (*p* < 0.05). On IHC, SHP2 was enriched in the epidermis in rosacea (*p* < 0.05), although 4G10 levels were not statistically significantly different between the two groups (*p* > 0.05).

**Conclusions:**

SHP2 is enriched in cutaneous specimens of rosacea, suggesting a critical role for this protein in the disease and indicating a modifiable therapeutic moiety.

## Graphical abstract

Rosacea is an inflammatory disorder with devastating secondary ocular surface effects. SH2 domain‐containing protein tyrosine phosphatase‐2 (SHP2) is enriched in rosacea samples, which furthers our understanding of its inflammatory pathogenesis. SHP2 is a potential target for future clinicians aiming to develop therapeutics for rosacea.

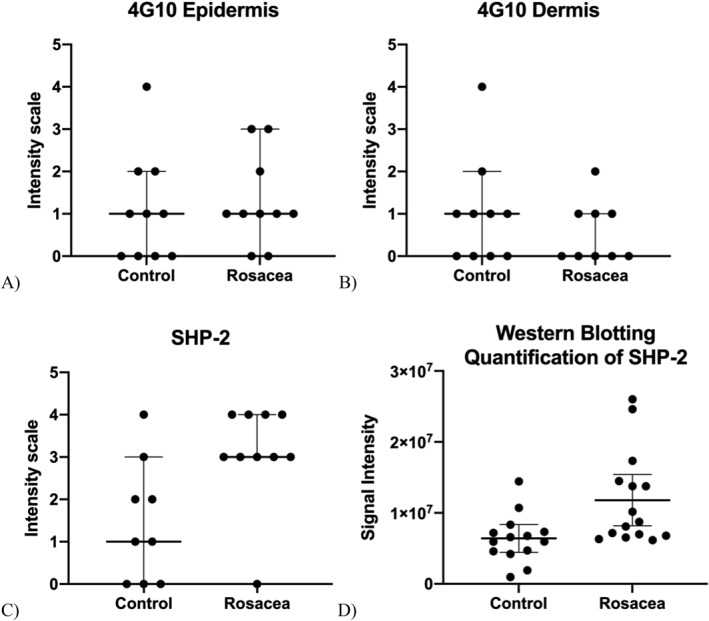

1

**What is already known about this topic?**

- Rosacea is an inflammatory disease with current treatments largely addressing the general inflammatory nature of rosacea and not the specific cellular and molecular changes that occur with the disease and are thus of limited utility and efficacy.

**What does this study add?**

- Our study further elucidates the inflammatory biochemical pathway for rosacea and identifies SH2 domain‐containing protein tyrosine phosphatase‐2 as a protein of interest for potential future intervention.

## 2 INTRODUCTION

Rosacea is a common disease that is estimated to affect 16 million Americans. Of these patients, 58%–72% develop ophthalmic manifestations including telangiectasias, ocular surface disease, conjunctival injection, meibomian gland destruction, and ocular discomfort.^1^ According to the National Rosacea Society, there was an initial classification system of rosacea which included several clinical subtypes (erythematotelangiectatic, papulopustular, phymatous, and ocular).^2^ A more recent classification is based on phenotypes rather than organizing these subtypes into distinct disorders.^3^ This classification system has organized the disease based on diagnostic and major criteria encompassing a multitude of possible combinations of signs and symptoms. Because ophthalmic manifestations of rosacea can occur without diagnostic skin findings, it can be underdiagnosed. Several diseases have been found to have statistically significant association with rosacea including depression, hypertension, cardiovascular disease, anxiety, dyslipidemia, diabetes mellitus, migraine, rheumatoid arthritis, *Helicobacter pylori* infection, ulcerative colitis, and dementia.^4^ Although causality between these disease associations and rosacea is not yet established, it is suspected that they all share a common immune, vascular, and inflammatory mediated pathogenesis. The American Academy of Dermatology estimates that rosacea results in $80 million in costs spent in health care annually.^5^ Understanding and developing therapeutics to this disease are crucial in reducing the vision threatening, cosmetic, psychological, and work productivity related impacts, and an improved comprehension of the cellular biology would facilitate novel treatment options. Wladis and associates have previously summarized various antibiotic, light based therapy, and surgical interventions that are currently available.^6^, ^7^ These current therapies largely address the general inflammatory nature of rosacea and not the specific cellular and molecular changes that occur with the disease and are thus of limited utility and efficacy. Previously implicated cellular features involving the immune system include toll‐like receptors, nuclear factor kappa‐B (NFKB), intracellular kinases (p38 and extracellular signal related kinase or ERK), myeloid differentiation factor 88 (MYD88), specific cytokines (interleukin‐1B and 16, stem cell factor, the monokine induced by interferon gamma [MIG/CXCL9], and macrophage chemoattractant protein‐1 [MCP‐1/CCL2]).^8^, ^9^, ^10^ This paper aims to expand our understanding of the cellular biology of rosacea by investigating the role of an intracellular phosphatase known as SHP2.

SH2 domain‐containing protein tyrosine phosphatase‐2 (SHP2, encoded by the PTPN11 gene) is a widely expressed cytoplasmic phosphatase that plays a key role in the development of various organs including the nervous system, the heart, the mammary gland, the kidney, and the intestines.^11^ Mutations in this gene and overexpression of SHP2 are associated with aberrations in a variety of cellular features that are associated with rosacea, including p38, ERK, and NFKB function.^12^ SHP2 has been found to play a critical role in the phosphorylation of p38 which in turn was critical for the phosphorylation of ERK in astrocytes.^13^ Downstream of the mitogen‐activated protein kinases (MAPK ‐ p38 and ERK) that are associated with rosacea, NFKB is a well‐known transcription factor that has been found to induce cytokine production and an inflammatory cascade.^14^ SHP2 has also been demonstrated to be an important cytoplasmic factor in efficient NFKB activation in fibroblasts in a MAP kinase independent fashion, suggesting multiple pathways for NFKB activation.^15^, ^16^ Taken together, the body of evidence from a variety of other diseases and cellular features strongly suggests that SHP2 activates a host of key cellular features that subserve rosacea. As such, we hypothesize that SHP2 may play a pivotal role in the pathogenesis of rosacea.

## 3 METHODS

This study was approved by the Institutional Review Board of Albany Medical College (#3581) and adhered to the tenets of the Declaration of Helsinki and the Health Insurance Portability and Accountability Act.

During patient recruitment, 20 patients with and without rosacea elected to undergo lower lid ectropion surgery and were identified from the practice of a single oculoplastic surgeon (EJW). These patients were diagnosed as having rosacea if they possessed a combination of classic examination features such as eyelid telangiectasias, blepharitis, conjunctival hyperaemia, and meibomian gland plugging on slit lamp examination.^17^ Immediately after resection, these specimens were frozen at −80℃ for use in western blotting (WB). An additional cohort of formalin‐fixed tissue biopsies were embedded in paraffin and used for immunohistochemical analysis. The immunohistochemistry (IHC) and WB protocols used in this study have been previously discussed by Wladis and associates and will be summarized here.^6^ The 4G10 antibody was used for comparison in IHC as its a commercially available phosphotyrosine antibody that is used to detect protein phosphorylation, which should be ubiquitously present in both the normal and rosacea phenotype.^18^ Thus, we hypothesize that there will not be a significant difference in expression between these phenotypes and that this will serve as a control for the isolation of SHP‐2.

### 3.1 Immunohistochemistry

The frozen samples were processed and embedded in paraffin sections. The slides were labelled 1–20 and were deparaffinized and rehydrated in xylene and ethanol (100% then 95% then 70%). Endogenous peroxidase activity was blocked with 0.5% H_2_O_2_/MeOH for 10 min at room temperature and antigen retrieval was done for 30 min at 100℃ in 10 mM citrate buffer, pH 6. Samples were blocked with 5% Foetal Bovine Serum for 1 h at room temperature. Primary antibodies against both 4G10 and SHP2 (Abcam) were incubated on separate sections at a dilution of 1:80 in phosphate‐buffered saline (PBS) overnight at 4℃. Biotinylated anti‐mouse secondary antibodies (Vector ba‐9200) 1:500 in PBS were incubated for 1 h at room temperature. On the slides where there were multiple areas of tissue present, a control area was set to ensure that staining was adequately performed. Samples were then incubated with avidin/biotin peroxidase (Vector ABC kit Elite PK‐6100) in the dark for 30 min at room temperature and the signal was detected with 3,3′‐diaminobenzidine (Immpact DAB, Vector SK‐4105). The slides were then counterstained with haematoxylin (Vector H‐3404) prior to dehydration and mounting with VectaMount (Vector H‐5000).

### 3.2 Western blotting

The frozen samples were also thawed and prepared for protein extraction. Specifically, 300 μl ice‐cold lysis buffer containing 1% Triton X‐100 (Sigma‐Aldrich) in PBS containing protease and phosphatase inhibitor cocktails (Roche) and 50 mM pervanadate (Sigma‐Aldrich) was added to the sample, together with 250 mg of zirconia/silica beads (Biospec Products Int). Then samples were homogenized through three cycles of 1 min each on a Mini‐Beadbeater‐96 (Biospec Products Int). Samples were centrifuged at >12 000 g for 2 min at 4 C and the supernatant was cleared by a second centrifugation for 15 min at >12 000 g for 2 min at 4 C. Lysates were aliquoted. These aliquots received 1× volume of 2× Laemmli buffer and were boiled for 5 min. A mix of equal parts of each lysate was run on each gel and used for normalization purposes.

Western blots were performed using 10 μl of lysate/lane and detected using antibodies against SHP2 (Abcam). Horseradish peroxidase–conjugated secondary antibodies were from Jackson ImmunoResearch. Signal was detected with Clarity Western ECL Substrate (Bio‐Rad) and a BioRad ChemiDoc MP imager. Band quantification was performed using BioRad ImageLab software from raw image files according to manufacturer's instructions.

### 3.3 Analysis

**FIGURE 1:**
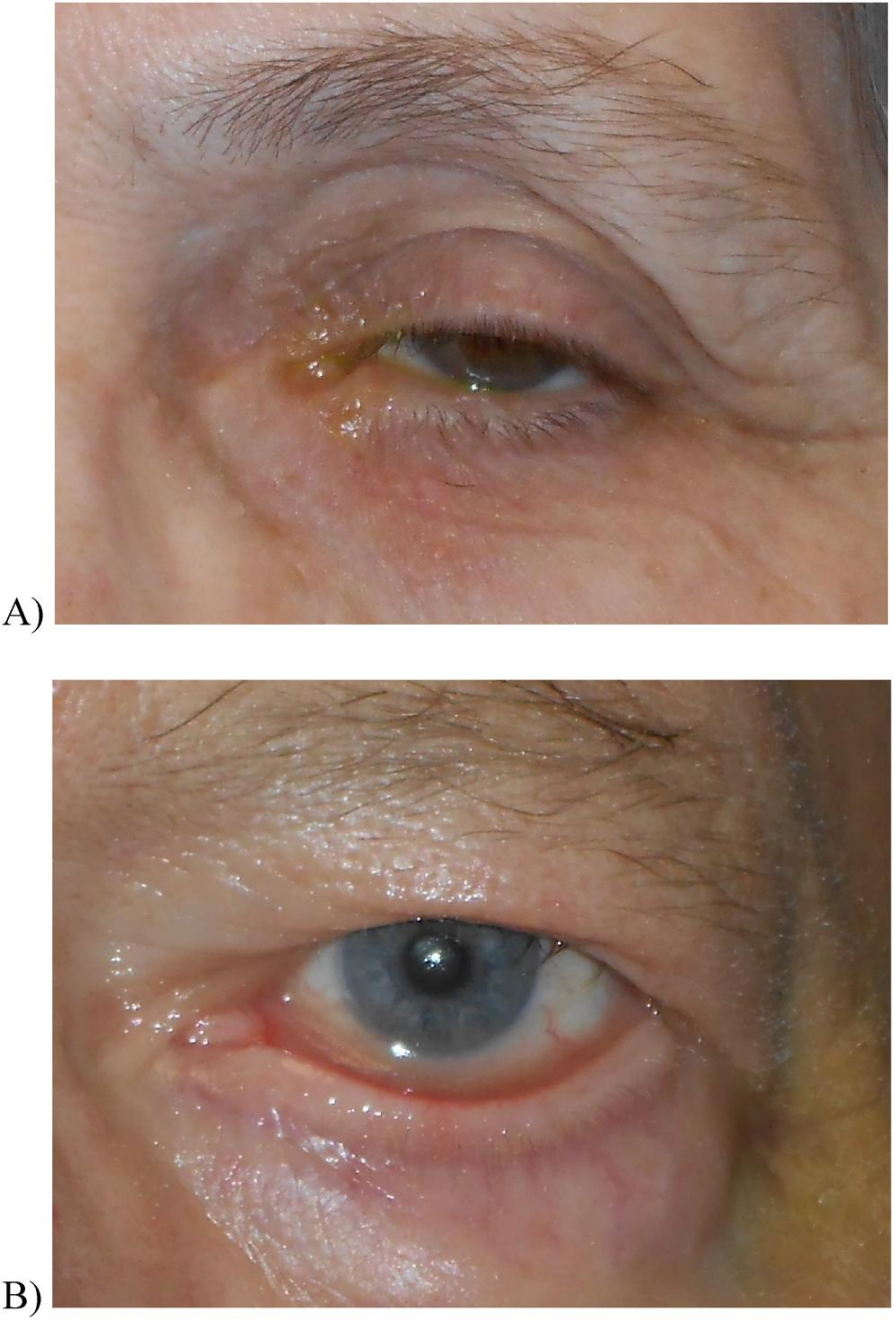
These photographs demonstrate the representative features present in patients with (a) a normal phenotype, lacking clinical features of rosacea, and (b) rosacea (telangiectasias, increased tear lake, ocular surface dryness).

Staining intensity grading was performed on all IHC specimens using a grading system of 0–4 (0 indicating no staining and four indicating the strongest possible staining) by two independent members of the research team (AC and RBR) to reach consensus. Patient information was deidentified and specimen diagnosis was masked during IHC, WB, and intensity grading. Microscope slide photographs for all IHC samples were obtained at 4×, 20×, and 40× magnification. For the Western blot experiments, normalized band intensity values (SHP2/actin) were used. After data collection was complete, specimens were unmasked, and Mann‐Whitney statistical analysis was performed using GraphPad Prism. Sample photographs for the normal and rosacea phenotypes were also taken for reference and will be included below in Figure 1.

## 4 RESULTS

Out of the 20 patient samples that were analyzed, 10 were the normal phenotype and 10 were rosacea phenotype. Patient demographics were similar between these two groups.

### 4.1 Immunohistochemistry

**FIGURE 2:**
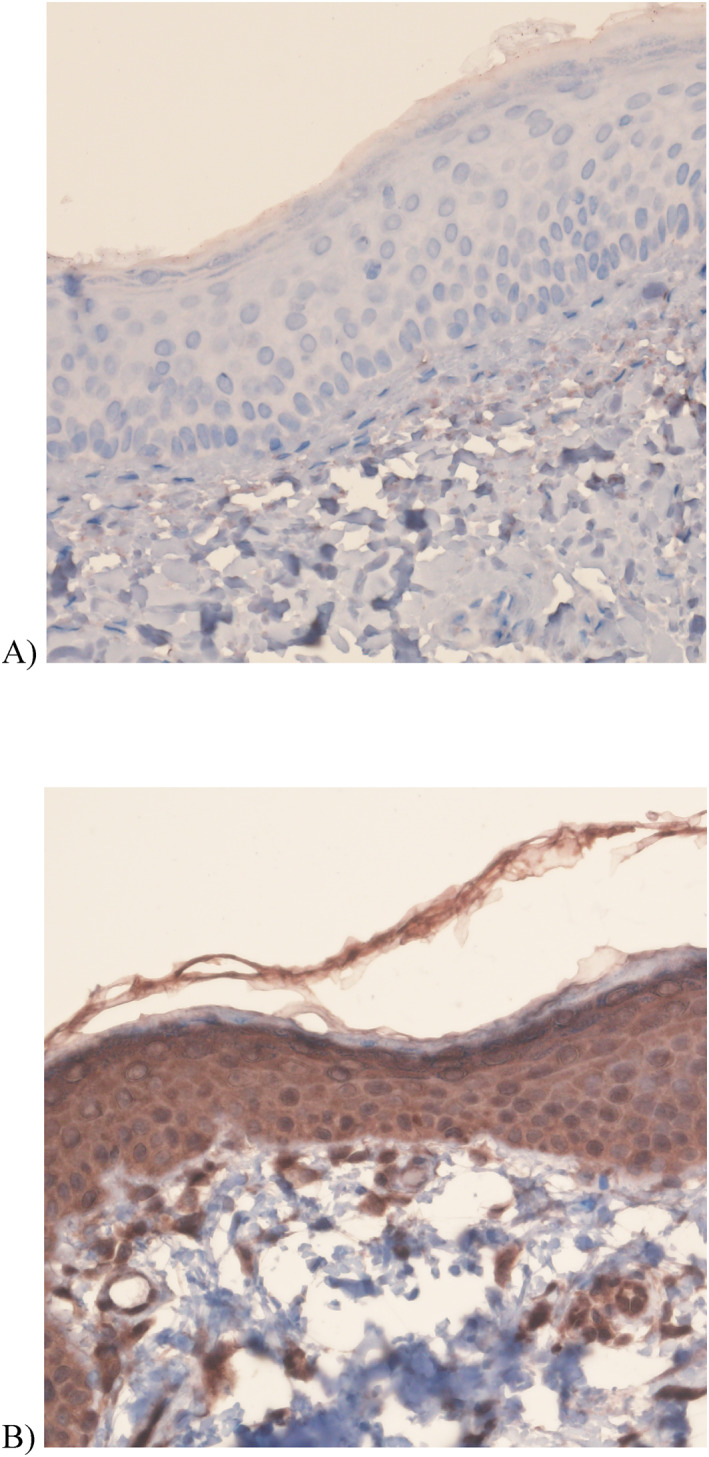
A representative photograph with 40× magnification demonstrates immunohistochemical staining with SH2 domain‐containing protein tyrosine phosphatase‐2 in (a) a patient with normal phenotype with minimal staining and (b) a patient with rosacea with intense staining in the epidermis.

When analyzing the slides with 4G10 staining, there was no statistically significant difference in intensity staining between control and rosacea samples in either the epidermis (mean of control = 1.1 and mean of rosacea = 1.3, *p* = 0.61) or dermis (mean of control = 1.0 and mean of rosacea = 0.55, *p* = 0.49). For the SHP2 slides, we observed an increase in intensity staining in rosacea samples primarily in the epidermis, as compared to control specimens and was statistically significant (mean of control = 1.4 and mean of rosacea = 3.1, *p* = 0.016). Figure 2 shows a representative photograph of the staining performed with 40× magnification.

### 4.2 Western blotting

**FIGURE 3:**
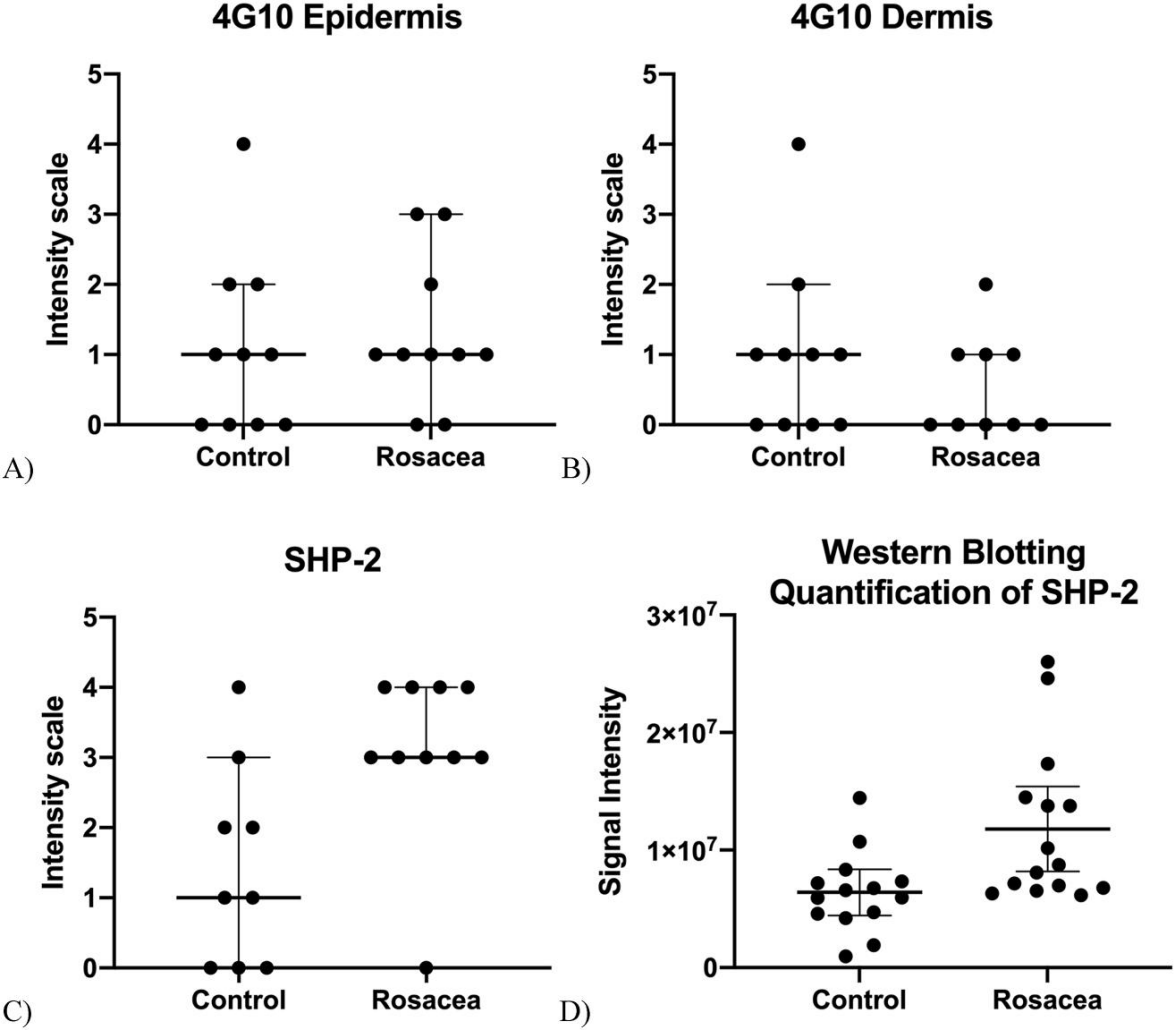
Immunohistochemistry (IHC) (a–c) performed shows (a) there is no significant difference in expression of 4G10 in the epidermis between control and rosacea (*p* > 0.05). (b) The same result is shown in the dermis for 4G10 between control and rosacea groups (*p* > 0.05). (c) However, there is an increased expression of SH2 domain‐containing protein tyrosine phosphatase‐2 (SHP2) localized to the epidermis in rosacea (*p* < 0.05). (d) Western blotting (WB) confirms that there is an increase in SHP2 in the rosacea samples (*p* < 0.05).

Western blotting performed on cutaneous eyelid samples showed a significant increase in signal band intensity of SHP2 in rosacea samples when compared with the control specimens (mean of control = 6 406 120.6 and mean of rosacea = 11 798 528.4, *p* = 0.008). The above results are summarized in Figure 3, which is a graphical representation of intensity in both control and rosacea in IHC and WB.

## 5 DISCUSSION

Compared with control specimens, SHP2 is enriched in cutaneous biopsies taken from patients with rosacea. Our testing with IHC and WB on two separate cohorts of patients confirms our hypothesis and implicates SHP2 in the pathogenesis of rosacea. As expected, SHP2 was primarily localized to the epidermis and 4G10 was expressed in both the epidermis and dermis.

Rosacea is primarily a cutaneous disease, which secondarily affects the ocular surface.^8^ Wladis and associates previously summarized the signalling pathways involving Toll‐like receptors and the inflammatory cascade that follows with NF‐kB and MAP kinases (Erk and p38). These inflammatory mediators were almost exclusively restricted to the epidermal layer of skin, which is similar to the distribution of SHP2 in the current investigation.

SHP2 has been connected as a key regulator of receptor tyrosine kinase and cytokine receptor signalling in addition to two multisystem developmental diseases (Noonan syndrome and LEOPARD syndrome).^19^ Recent literature has emerged discussing the anti‐tumour effect of SHP2 inhibitors, which provides insight into the role SHP2 plays in multiple systemic disorders involving inflammation and tumourigenesis. SHP2 critically activates multiple cell signals and processes, including NF‐kB, nuclear factor of activated T‐cells, mTOR, ERK 1/2 and eventual T‐cell activation involving PD‐1, CTLA‐4, BTLA, TIGIT, ZAP70, CD28, and PI3K‐AKT signalling pathways. SHP2 has also been involved in the proliferation of macrophages as well. Using the tumour model for the interplay of SHP2 with the immune system, we propose a similar mechanistic juxtaposition of SHP2 with the inflammatory model in rosacea.

In another review, SHP2 has been proposed as a therapeutic target for multiple systemic inflammatory diseases.^20^ In the nervous system, SHP2 has been found to promote inflammation by increasing the production of CXCL‐8 (IL‐8), which has been linked to Alzheimer's disease through regulation of P38 and ERK. In the development of ulcerative colitis, SHP2 is involved in the activation of lipopolysaccharide (LPS) and Toll‐like receptor 4 signalling in macrophages leading to continuous activation of NF‐kB signalling transduction and worsening colitis.^21^, ^22^ A colitis protective effect has been proposed with the inhibition of SHP2. In the urinary system, LPS has been associated with the increase of inflammatory cytokines and SHP2 including the activation of NF‐kB and MAPK signalling pathways. A deletion of SHP2 has been associated with attenuation of these systemic inflammatory markers. In a mouse model of the respiratory system, cigarette smoke has been affiliated with an increase in SHP2 activity in pulmonary epithelial cells. Selective inhibition of SHP2 in these mice exposed to cigarette smoke showed a decrease in pulmonary inflammation and reduced IL‐8 release suggesting a pathogenetic role of SHP2 in cigarette smoke mediated alteration of lung tissue.^23^ Smoking has also been shown to enhance NF‐kB activity.^24^, ^25^

By successfully implicating the enrichment of SHP2 in rosacea, we propose that SHP2 is likely involved in the innate immune system and the resulting inflammatory cascade for rosacea specifically involving p38 and efficient NF‐kB activation. In using the previously discussed models of inflammation, cigarette smoke (a known exacerbator of rosacea) is likely also contributory in increased SHP2 levels in patients with rosacea.

The strengths of our study include the initial blinding to the diagnosis when performing the data collection during IHC, WB, and intensity grading. This helped to prevent bias while performing the laboratory protocols and when scoring samples. Another strength is our use of multiple confirmatory tests to confirm the overexpression of SHP2 in rosacea samples, which provides a multimodal avenue of supporting evidence. Finally, the presence of similar results in two cohorts of patients with appropriate statistical analyses ensures the fidelity of these findings. The major limitation in our study involved the use of a grading system based on intensity. However, adequate efforts were taken to minimize subjective grading by following an independent grading approach. Another limitation of our study is that enroled patients had a diagnosis of having rosacea solely based on clinical judgement. This limitation may be important in cases of subclinical rosacea which do not show those ophthalmic manifestations defined above. A third limitation of our study is the small sample size in each cohort. However, despite these limitations our study results still show statistically significant differences in SHP‐2 expression to implicate its role in rosacea.

As previously discussed, SHP2 has been implicated in multiple pathways for NFKB activation and inhibition of this protein may be critical in preventing an inflammatory cascade. In the future, clinicians should investigate SHP2 as a target for therapeutic intervention, which may provide relief for patients afflicted with rosacea.

## CONFLICT OF INTEREST

Alejandro Pablo Adam and Edward J. Wladis disclose a patent for the treatment of rosacea with p38 and Erk kinase pathway inhibitors (WO2017192928A1) and interest in Praxis Biotechnology, Inc. The authors have no other conflicts of interest to declare.

## AUTHOR CONTRIBUTIONS

**Apoorv Chebolu**: Conceptualization (Equal); Data curation (Equal); Formal analysis (Equal); Funding acquisition (Equal); Investigation (Equal); Methodology (Equal); Project administration (Equal); Software (Equal); Validation (Equal); Writing – original draft (Equal); Writing – review & editing (Equal). **Ramon Bossardi Ramos**: Data curation (Equal); Methodology (Equal); Writing – review & editing (Equal). **Thilaka Arunachalam**: Data curation (Equal); Methodology (Equal); Writing – review & editing (Equal). **Alejandro Pablo Adam**: Conceptualization (Equal); Methodology (Equal); Supervision (Equal); Writing – review & editing (Equal). **Edward J. Wladis**: Conceptualization (Equal); Formal analysis (Equal); Funding acquisition (Equal); Methodology (Equal); Supervision (Equal); Writing – review & editing (Equal).

## ETHICS STATEMENT

This study was approved by the Institutional Review Board of Albany Medical College (#3581) and adhered to the tenets of the Declaration of Helsinki and the Health Insurance Portability and Accountability Act.

## Data Availability

The data that support the findings of this study are available from the corresponding author upon reasonable request.
